# Gender Risk Perception and Coping Mechanisms among Ghanaian University Students during the COVID-19 Pandemic

**DOI:** 10.3390/healthcare10040687

**Published:** 2022-04-06

**Authors:** John Elvis Hagan, Frank Quansah, James Boadu Frimpong, Francis Ankomah, Medina Srem-Sai, Thomas Schack

**Affiliations:** 1 Department of Health, Physical Education and Recreation, University of Cape Coast, Cape Coast PMB TF0494, Ghana; james.frimpong@stu.ucc.edu.gh; 2 Neurocognition and Action-Biomechanics-Research Group, Faculty of Psychology and Sports Science, Bielefeld University, Postfach 10 01 31, 33501 Bielefeld, Germany; thomas.schack@uni-bielefeld.de; 3 Department of Educational Foundations, University of Education, Winneba P.O. Box 25, Ghana; fquansah@uew.edu.gh; 4 Department of Education and Psychology, University of Cape Coast, Cape Coast PMB TF0494, Ghana; francis.ankomah@stu.ucc.edu.gh; 5 Department of Education, SDA College of Education, Asokore-Koforidua P.O. Box AS 18, Ghana; 6 Department of Health, Physical Education, Recreation and Sports, University of Education, Winneba P.O. Box 25, Ghana; mssai@uew.edu.gh

**Keywords:** coping, COVID-19, gender, Ghana, risk perception, university students

## Abstract

Recent research has shown that gender is an important driver of the risk of mortality and morbidity rates for people with COVID-19, with case fatality rates being higher for women than men. Despite this pattern, research is sparse on gender risk perception and potential coping mechanisms. This study examined the role gender plays in the relationship between COVID-19 risk perception and coping mechanisms among university students. Through the adoption of traditional and online surveys, 859 students from two public universities in Ghana were conveniently selected to respond to the survey instrument. The results from the multivariate regression analysis revealed that COVID-19 risk perception was positively related to active coping. The outcome of the moderation analysis showed that while males were more likely than females to adopt active and emotional support coping with heightened risk perception, a contrary outcome was observed for behaviour disengagement. This result is an indication that female students are likely to be overwhelmed with a high level of risk perception and easily give up trying to adopt effective strategies to reduce the effect of the COVID-19 pandemic situation. The findings highlight the need for different forms of intervention for male and female students for dealing with the effect of COVID-19.

## 1. Introduction

The spread of the coronavirus-2019 disease (COVID-19) intensified around the globe since its outbreak. The global situation of COVID-19 confirmed cases as of 4 February 2022 stood at 386,548,962, with 5,705,754 cumulative deaths [1]. The WHO also reported that a total of 10,040,768,270 vaccine doses had been administered worldwide as of 2 February 2022 [1]. Ghana’s situation regarding the COVID-19 pandemic as of 4 February 2022 stood at 156,920 confirmed cases with 1395 deaths [2]. It has also been reported that 9,745,332 vaccine doses had been administered as of 2 February 2022 [2]. All of these figures are enough to trigger unbearable psychological ramifications. Recent research has indicated the long-lasting effects and severity of psychological conditions resulting from lockdowns, quarantines, and isolations during COVID-19 [3]. The spread of negative COVID-19 related information on social media also increases persistent and recurrent feelings of worry [4]. Furthermore, research has proven that the COVID-19 outbreak increases the magnitude of anxiety, depression, and susceptibility to social risks, which in turn decrease life gratification and positive emotions [5,6].

The transition from lockdowns, quarantines, and isolations to vaccination and injections of COVID-19 boosters may cause people, including university students, to be worried, feel anxious, and perceive themselves as being at risk of contracting the COVID-19 virus. Perceived risk is defined as an individuals’ psychological evaluation of the likelihood and consequences of a hostile outcome [7]. One of the important factors that cause people to willingly engage in health-protective behaviours is risk perception. Essentially, one’s personal understanding of risky occurrences can direct their health behaviours towards unanticipated hazards like COVID-19 [8]. In this light, people have the ability to adapt to situations they perceive as risky and have detrimental effects on them [8]. For instance, people express their risk perception by engaging in precautionary behaviours such as avoiding crowded public events, practising social distancing, and practising personal hygiene [9]. Research has also revealed that perceived risk during a pandemic is related to anxiety, disruptions in daily routines, worry [3,8,10,11,12,13], and coping strategies [11,14,15]. However, whether or not there is any linkage between perceived risk and coping mechanisms during COVID-19 among Ghanaian university students is not documented, creating a void in the literature that needs urgent attention. Previous literature has suggested the effect of pandemics on students’ psychology [16], with university students being at greater risk for anxiety and depression during such outbreaks [17,18]. Other COVID-19 studies have already shown that students’ stress, anxiety, and depression experiences deteriorated compared to reported levels prior to the pandemic [19,20]. Hence, investigating university students’ risk perception across gender and potential coping strategies may provide university authorities with useful information to guide or plan and implement appropriate interventions aimed at promoting students’ mental health and well-being.

According to the protection-motivation theory, people are more likely to protect themselves in line with their perception of the severity of forthcoming threatening events, their perceived vulnerability and self-efficacy [21,22]. As a result of perceived heightened risk during pandemics such as COVID-19, people analyse the costs and benefits of certain precautionary actions before engaging in them [23,24]. Considerable levels of perception of risk are critical for people to effectively battle the pandemic and devise efficient preventive health behaviours, but a reduced level of risk perception of infection would defeat such an aim [25]. For instance, people who perceive themselves to be at a high risk of contracting a disease like COVID-19 may follow the recommended preventive protocols than people who do not perceive themselves to be at risk [25].

Gender has been identified as an important driver of risk for mortality and morbidity rates for people who have been struck by COVID-19 [8]. Available data indicate that rates of mortality resulting from COVID-19 are higher for women than men [26,27]. Other research works have also revealed that risk perception for COVID-19 is higher among men compared with their female peers [28,29]. Meanwhile, other studies have also found that women have a higher risk perception than men [24,30,31]. These results suggest that the extant literature is fraught with inconsistencies that need to be resolved, thus forming one of the objectives of this study. Previous studies linked to COVID-19 have focused on themes like epidemiology, genetic characteristics of the virus, and its clinical consequences [32,33]; however, there is sparse information on the COVID-19 pandemic-related gender variations on risk perception and associated coping mechanisms among students in Ghana. The only documented study that examined the role of gender in risk perception and coping was conducted in Pakistan and was restricted to the general population [34]. This limits the generalization of the findings reported in previous studies in other jurisdictions [30]. Investigating students’ risk perceptions may be key for emergency management during public health emergencies like COVID-19 [35]. Burn and Slovic [35] reiterated that public risk perceptions usually generate attitudes, emotional reactions, and prevention behaviours, including disease control strategies. For instance, the effect of a high level of perceived risk among Ghanaian university students has been demonstrated in recent research by Quansah et al. [36]. The authors [36] revealed that students who reported high level of risk perception exhibited a high level of anxiety, and were likely to adopt a high level of behaviour disengagement coping (i.e., maladaptive).

Therefore, this study examines the influence of COVID-19 risk perception and coping mechanisms, as well as the role of gender in the relationship between COVID-19 risk perception and coping mechanisms, using a sample of Ghanaian university students. This study has great significance for virus control in educational institutions in the country. The findings could help shape policies and interventions (e.g., appropriate risk communication messages) that may lead to compliance with COVID-19 preventive and management protocols and also promote a safe school environment conducive for learning. Internationally, this study serves as a reference for future COVID-19 studies that focus on risk perception and coping mechanisms.

## 2. Materials and Methods

### 2.1. Study Context and Sample Characteristics

Through a cross-sectional survey, 859 students were sampled from two public Ghanaian universities (i.e., University of Education, Winneba, UEW, and University of Cape Coast, UCC). The reference population for this study was university students in Ghana. However, the accessible population was limited to students in the two universities (i.e., UEW and UCC) due to the following reasons: (1) Observations made by the investigators as they went about their teaching activities during the COVID-19 pandemic showed that students in these two universities reportedly showed (anecdotal evidence) some level of fear, worry, and uncertainty regarding their safety during teaching and learning activities. Thus, the investigators believed that such a group of students could provide adequate information about the phenomenon being studied. (2) Empirical evidence from two recent studies in the same universities using physical education students also showed that the level of risk perception was on the rise [15,36], and therefore, this group was believed to provide useful and interesting results.

The sample size for this research was estimated using G-power software with an effect size of 0.02, power (1-*β*) estimate of 0.95, α error probability of 0.05, and five predictors (including interaction term). This estimation procedure yielded a sample size of 995. However, 136 participants either opted out of the study or did not complete about 90% of the items on the questionnaire, leading to a response rate of 86%. A convenient sampling procedure was used to select the students to participate in the study; participants who were ready, willing, and available at the time of data collection were recruited to respond to the data collection instrument. This sampling approach was used due to the non-accessibility to the sampling frame for the accessible population. Out of the 859 students, 36.2% were below 23 years (*n* = 311), 47.8% were aged 24 to 30 (*n* = 410), and the rest were over 30 years old. The study participants were dominated by males (*n* = 536, 62.4%), with females constituting 37.6% (*n* = 323). Christians constituted about two-thirds of the sample (*n* = 581, 67.6%), Muslims represented about 26.5% (*n* = 228), whereas the African tradition religion represented 0.7% (*n* = 6).

### 2.2. Study Measures

#### 2.2.1. Risk Perception

The risk perception index was computed to reflect the degree of COVID-19 risk perceived by the participants. Risk perception was conceptualized as the extent to which the participants perceived their environment (university campus) as risky in terms of contracting and transmitting COVID-19 infections. A set of five items were carefully adapted from Capone et al.’s [37] COVID-19 Risk Perception (CoRP) scale. The participants were required to indicate the extent to which they perceived their environment safe from COVID-19 infections. The items were measured on a “yes” or “no” response format. The items were as follows: “It is very easy to contract the COVID-19 virus within the school environment”, “I know of colleagues who have contracted the virus and still going about their normal activities”, “I fear talking to colleagues because I am likely to be infected when I do that”, “I am uncertain about the safety of the school environment”, and “I am at a high possibility of contracting the virus with the least mistake I make”. For each item, a “yes” response was scored 1 and a “no” response was scored 0. For easy interpretation, the composite was scored out of 5 and was converted to an index that ranged from 0 to 1 by dividing the participant’s total score by 5. The Response Factor Analysis (RFA) was employed to calibrate the COVID-19 Risk Perception Scale, showing factor loadings between 0.67 to 0.88. Using the Kuder–Richardson reliability estimate [38,39], a reliability estimate of 0.705 was achieved, which was deemed sufficient [40].

#### 2.2.2. Coping Mechanism

The coping inventory for university students developed and validated by Quansah et al. [41] was adopted for this study. The inventory was multidimensional with four subscales, namely, active coping, behaviour disengagement, religious coping, and emotional support. Each dimension had four items measuring the construct. The items included, “I take additional action to try to get rid of the problem”, “I just give up trying to reach my goal because of the stressor”, and “I just give up trying to reach my goal because of the stressor”. The Omega ω reliability estimates reported by the original developers were 0.823 for active coping, 0.869 for behaviour disengagement, 0.812 for religious coping, and 0.826 for emotional support. This study reported the following Omega ω reliability coefficients for the scales: active coping 0.816, religious coping 0.783, behaviour disengagement 0.843, and emotional support 0.821. These indices were deemed sufficient [38,40].

### 2.3. Procedure

With approval from the Institutional Review Board (IRB) of the University of Cape Coast, with reference number UCCIRB/EXT/2020/25, data collection commenced in the two public universities selected. With the help of some heads of departments and faculty members, students were sensitized regarding the upcoming data collection exercise. The data collection was carried out using two approaches: traditional face-to-face administration and online administration. Respondents had the opportunity to choose their preferred mode of administration. With the online mode, respondents were required to provide identification indicators (like programs, hall of affiliation, and affiliated faculty) to authenticate that they were students and were eligible to be part of the study. Meanwhile, links were only shared to targeted participants through emails, Telegram, and WhatsApp platforms. The traditional mode of administration was conducted in the lecture halls where copies of the questionnaire were distributed to students through a convenient sampling technique. Throughout the questionnaire administration period, the investigators adhered to ethical standards such as consent, anonymity, confidentiality, protection from emotional harm or discomfort, protection of vulnerable participants, volition, and freedom of withdrawal. For example, participants signed a consent form to declare their endorsement to be part of the study. In addition, none of the information sought from the participants could reveal their identity. The respondents were made aware that they could freely withdraw from responding to the instrument at any point in time without any reason. The entire data collection was done in 2021 and lasted for 2 to 3 months.

### 2.4. Data Analyses

The data collected were checked for correctness and appropriateness before performing the analysis. All of the analyses were performed using SPSS (version 25). The data were first explored to understand the nature of the variables, in terms of the means and standard deviations, as well as the correlations existing among the key variables of the study. Using the multivariate linear regression, the relationship between COVID-19 risk perception and coping mechanisms was examined. A stringent alpha was set to 0.013 (based on the four dependent variables) for this analysis in order to reduce the effect of Type 1 errors. Furthermore, simple moderation analysis was carried out to test the moderating effect of gender in the relationship between COVID-19 risk perception and coping mechanisms. The moderation analysis was carried out using 5000 bootstrap samples and, thus, the bootstrap confidence interval was used for the interpretation. The PROCESS macro by Hayes (add-on in SPSS) was used for the moderation analysis using the Model 1 function. The effect size estimations were computed for all of the inferential analyses conducted using Cohen’s *f*^2^ with the following cut-off points: 0.02 for small, 0.15 for medium, and 0.35 for large effect size.

## 3. Results

### 3.1. Preliminary Analyses on Risk Perception, Coping Mechanisms, and Gender

The descriptive analyses of the variables have been presented in Table 1.

Risk perception, based on the index, showed a high level of risk perception among the participants (*M* = 0.733, *SD* = 0.223) (see Table 1). The variable risk perception was found to be significantly related to active coping (*r* = 0.173) and gender (*r* = −0.125). The analyses showed that religious coping was the top utilized coping strategy (*M* = 2.79, *SD* = 0.860), followed by active coping (*M* = 2.64, *SD* = 0.755) and emotional support (*M* = 2.50, *SD* = 0.71). Religious coping was significantly related to behaviour disengagement (*r* = 0.204), emotional support (*r* = 0.339), and gender (*r* = 0.078). Behaviour disengagement was also significantly related to emotional support (*r* = 0.325) and gender (*r* = −0.138).

### 3.2. Influence of COVID-19 Risk Perception on Coping Mechanism

The study examined the influence of COVID-19 risk perception on coping mechanisms. The outcome of the multivariate linear regression analysis is shown in Table 2.

As shown in Table 2, the COVID-19 risk perception of students positively predicted active coping, *B* = 0.573, *SE* = 0.111, *t* = 5.138, *p <* 0.001, *f*^2^ = 0.031, indicating that the more university students perceived their environment as being risky in terms of contracting COVID-19, the more they utilized active coping mechanisms. Risk perception failed to significantly predict the other three dimensions of coping mechanisms, namely: religious coping, behaviour disengagement, and emotional support.

### 3.3. The Role of Gender in the Relationship between COVID-19 Risk Perception and Coping Mechanism

Through a single moderation analysis, the role of gender in the relationship between COVID-19 risk perception and coping mechanism was explored. The details of the results are shown in Table 3.

**Figure 1:**
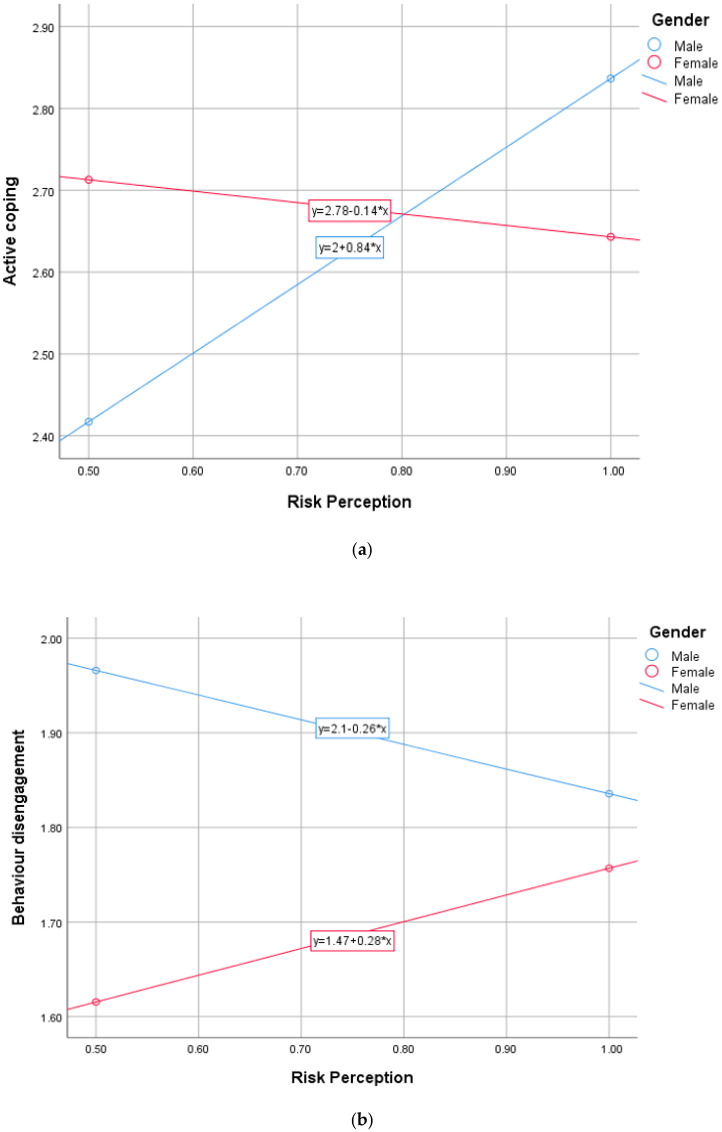

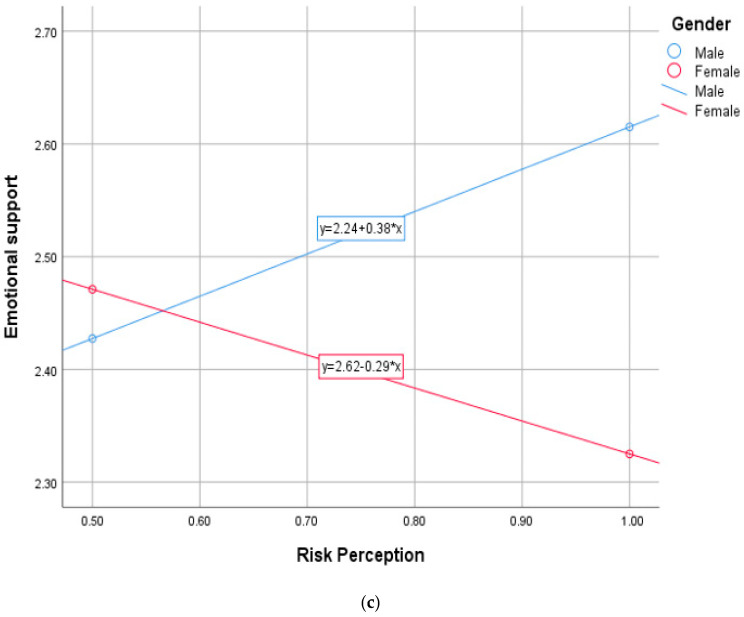
(**a**) Moderation effect of gender in the relationship between COVID-19 risk perception and active coping. (**b**) Moderation effect of gender in the relationship between COVID-19 risk perception and behaviour disengagement coping. (**c**) Moderation effect of gender in the relationship between COVID-19 risk perception and emotional support coping.

In Model 1, the results showed that gender significantly moderated the relationship between risk perception and active coping, *B* = −0.979, *t* = −3.795, *BootCI*(−1.485, −0.473), *f*^2^ = 0.175 (see Table 3). Probing the analysis showed that in the midst of a high level of risk perception, male students adopted active coping to a greater extent (see Figure 1a). Meanwhile, female students utilized less active coping. The results in Model 3 revealed a significant moderation of gender in the relationship between risk perception and behaviour disengagement, *B* = 0.544, *t* = 2.146, *BootCI* (0.046, 1.041), *f*^2^ = 0.144. Female students adopted high levels of behaviour disengagement strategy when risk perception was high, whereas male students adopted this coping mechanism to a very low extent in the same situation (see Figure 1b). The results (in Model 4) also showed a significant moderating effect of gender in the relationship between risk perception and emotional support, *B* = −0.668, *t* = −2.701, *BootCI* (−1.153, −0.182), *f*^2^ = 0.202. The results suggested that in the presence of a high level of risk perception, male students adopted more emotional coping, while female students utilized less emotional coping (see Figure 1c). However, gender did not moderate the relationship between COVID-19 risk perception and religious coping.

## 4. Discussion

The core focus of this study was to ascertain the influence of COVID-19 risk perception and coping mechanisms, taking into consideration gender variations among university students, and how likely they are confounding the influence. The findings indicated heightened risk perception among university students. Religious coping was predominantly adopted as a coping mechanism by the students, while behaviour disengagement was the least adopted coping mechanism. Among the four dimensions of coping strategies used in this study, COVID-19 risk perception influenced only active coping. Thus, a high level of risk perception was associated with the increased practice of active coping mechanisms with a positive influence. This finding signifies that the mere perception of the university environment as being unsafe in terms of the transmission and contracting of COVID-19 is associated with high chances of students wearing nose masks, and also adhering to hygiene and management protocols. In line with this finding, Yıldırım et al. [12] documented that in expressing risk perception, people engage in precautionary behaviours such as avoiding crowded public events, practising social distancing, and practising personal hygiene. These precautionary behaviours are practical as they serve as a barricade to the transmission and contracting of COVID-19 disease. The aforementioned are some preventive measures outlined by the WHO in response to the COVID-19 pandemic. Yıldırım and Güler [8] noted that when people weigh the outcome of contracting COVID-19 as being devastating, that alone propels them to adopt preventive behaviours. Other studies have equally established a link between the perception of risk associated with COVID-19 and coping strategies [10,13,14].

The findings of the current study can be situated within the framework of protection motivation theory [22]. This theory posits that one’s personal motivation for protection triggers their engagement in particular behaviours vis-à-vis health treats. In this regard, risk perception can be likened to students’ personal motivation for protection against COVID-19, while their engagement in particular behaviours has to do with their adoption of coping mechanisms. Therefore, students are more likely to protect themselves in line with their perception of the severity of forthcoming threatening events that could emanate from COVID-19 by way of adopting active coping mechanisms that are preventive.

The findings further established that, with the introduction of gender, three of the four coping mechanisms, namely, active coping, behaviour disengagement coping, and emotional support coping, were influenced by risk perception. Meanwhile, only active coping had a direct influence on risk perception without the presence of gender in the model. These findings underscore the idiosyncratic nature of gender in the relationship between risk perception and coping mechanisms. While males were more likely than females to adopt active coping and emotional support coping with heightened risk perception, the relationship was otherwise in the case of behaviour disengagement. This was supported in a study by Alsharawy [34], who found that women compared to men reported high rates of negative emotions. Rana et al. [31] also reiterated that women exhibited higher levels of fear than men. The implications of the findings are that, with an increased level of risk perception, males take pragmatic steps in dealing with the problem, and sometimes, they derive sympathy and support from friends and relatives as ways of coping. Females rather prefer to quit or give up via disengagement, suggestive of avoidant coping mechanisms (i.e., maladaptive), because they cannot probably deal with situations perceived as threatening. Reflecting on these results, a possible explanation could be that males are naturally seen to be resilient to traumatic and stressful situations, hence their adoption of adaptive coping strategies. This could explain why the males who adopted the aforementioned coping strategies adapted to the adverse conditions when they perceived high risk of COVID-19 transmission and possible infection [9,42]. Comparatively, females were quite susceptible to stressful situations, and hence, were less likely to adopt active coping strategies in the face of life adversities during emergencies [9,43].

The findings have empirical underpinnings from previous researchers [11,24,26,27,28,29,30]. For example, Cai [26], and Wu and McGoogan [27], found that the severity of COVID-19 and its death toll was on the high side for females compared with males. In relation to the current study, while females become much more aware of the severity and the death toll, perceived themselves as being more susceptible to COVID-19. Consequently, females were more likely to adopt avoidance coping mechanisms (e.g., behaviour disengagement) because of high COVID-19 risk perception [11,24,26,27,30]. According to Maiorano et al. [44], feeling a sense of helplessness and inability may compel female students to exhibit the adoption of non-proactive attitudes through the use of avoidance coping strategies as contagion measures. Furthermore, under high risk perception, females compared to their male counterparts have also been suggested to be more affective than cognitive by some researchers, from the affect heuristic perspective. The same justification could hold for the variations noted across gender in utilising varied coping strategies [34]. From the abovementioned, it is clear that gender may confound the direct relationship between COVID-19 risk perception and coping mechanisms. This informs gender-specific public health interventions in terms of risk perception and coping.

### Limitations

The present study has some limitations. The study used a cross-sectional design, collecting data from at a single point in time during the pandemic; hence, conclusions drawn from the findings do not guarantee long-term effects. Future studies could use longitudinal studies to evaluate changes in COVID-19 risk perception and coping mechanisms to mirror the dynamic nature of the pandemic using other student populations in the country. Furthermore, participants in this research were selected through a convenience sampling technique and this could provide a biased sample. The generalization of findings to other student populations could be limited. Despite these limitations, the findings provide useful information to key stakeholders of university campuses in Ghana to implement health promotion and prevention interventions essential to boost successful adaptation, especially among females in this challenging pandemic era.

## 5. Conclusions

The import of our research emphasized the relationship between COVID-19 risk perception and coping mechanisms, addressing the role that gender plays. It was clear that as university students experienced high COVID-19 risk perception, these students were found to adopt an active coping mechanism. This notwithstanding, this relationship existing between COVID-19 risk perception and coping mechanisms appeared not to be the same for both male and female students. In the presence of a high level of risk perception, male students largely adopted the active and emotional coping mechanisms, whereas female students utilized less of these coping strategies. However, female students largely adopted behaviour disengagement coping amid high COVID-19 risk perception. The result is an indication that female students are likely to be overwhelmed with a high level of risk perception and give up easily trying to put in place effective strategies to reduce the effect of the COVID-19 pandemic situation. The findings of the study highlight the need for different forms of intervention for male and female students in dealing with the effect of COVID-19. This research discourages the implementation of wholesale COVID-19 intervention strategies in schools. That is, appropriate intervention packages need to be designed and implemented for students, taking into consideration the diverse ways in which each group responds to the pandemic situation. University students should be sensitized to the risks associated with COVID-19, including varied risk perception and the adoption of active coping strategies, with special emphasis on female students.

## Figures and Tables

**Table 1:** Descriptive statistics for the study variables.

No.	Variables	1	2	3	4	5
1	Risk perception	1				
2	Active coping	0.173 **	1			
3	Religious coping	0.053	0.406 **	1		
4	Behaviour disengagement	−0.021	0.143 **	0.204 **	1	
5	Emotional support	0.074 *	0.410 **	0.339 **	0.325 **	1
6	Gender ***	−0.125 **	0.035	0.078^*^	−0.138 **	−0.063
-	Mean	0.733	2.641	2.790	1.843	2.50
-	SD	0.228	0.755	0.860	0.733	0.711

* significant at *p* < 0.05; ** significant at *p* < 0.01; *** Gender was coded as 1–female, 0–male.

**Table 2:** Regression parameters for the relationship between COVID-19 risk perception and coping mechanisms.

Criterion Variable	Parameters	*B*	Std. Error	*t*	*p*	*f* ^2^
Active coping	Intercept	2.222	0.086	25.970	0.000	0.031
	Risk perception	0.573	0.111	5.138	0.000 *	
Religious coping	Intercept	2.645	0.099	26.763	0.000	0.003
	Risk perception	0.198	0.129	1.541	0.124	
Behaviour disengagement	Intercept	1.893	0.084	22.460	0.000	0
	Risk perception	−0.069	0.110	−0.628	0.530	
Emotional support	Intercept	2.326	0.082	28.491	0.000	0.005
	Risk perception	0.231	0.106	2.174	0.030	

* significant at *p* ≤ 0.013.

**Table 3:** Moderation parameters for gender in the link between COVID-19 risk perception and coping mechanisms.

Models		*B*	*SE*	*t*	LLCI	ULCI	*f* ^2^
1	Constant	1.998	0.101	19.877	1.800	2.195	0.175
	Risk perception (RP)	0.839	0.128	6.535	0.587	1.091	
	W1	0.785	0.190	4.137	0.413	1.158	
	RP*W1	−0.979	0.258	−3.795	−1.485	−0.473	
2	Constant	2.489	0.117	21.333	2.260	2.718	0.013
	Risk perception (RP)	0.350	0.149	2.351	0.058	0.643	
	W1	0.486	0.220	2.203	0.053	0.918	
	RP*W1	−0.451	0.400	−1.506	−1.039	0.137	
3	constant	2.096	0.099	21.235	1.903	2.290	0.144
	Risk perception (RP)	−0.261	0.126	−2.069	−0.509	−0.013	
	W1	−0.623	0.187	−3.338	−0.989	−0.256	
	RP*W1	0.544	0.253	2.146	0.046	1.041	
4	Constant	2.240	0.096	23.259	2.051	2.429	0.202
	Risk perception (RP)	0.375	0.123	3.051	0.134	0.617	
	W1	0.378	0.182	2.076	0.021	0.735	
	RP*W1	−0.668	0.247	−2.701	−1.153	−0.182	

Outcome variables: Model 1—active coping; Model 2—religious coping; Model 3—behaviour disengagement; Model 4—emotional support; W1—female (with male as a comparison group).

## Data Availability

The data are available upon reasonable request from the corresponding author.
